# Supplementary material for: Gut microbiota mediates intermittent-fasting alleviation of diabetes-induced cognitive impairment
Source: Nat Commun. 2020 Feb 18;11:855. doi: 10.1038/s41467-020-14676-4 (PMC7029019; doi:10.1038/s41467-020-14676-4)
Supplement: Supplementary file 1 — Supplementary Information [file 41467_2020_14676_MOESM1_ESM.pdf]

Supplementary Information for

Gut Microbiota Mediates Intermittent-Fasting Alleviation of Diabetes Induced Cognitive Impairment

Zhigang Liu et al.

Supplementary Methods

Elevated plus maze test

The elevated plus maze test is a well-established model used to evaluate anxiety behavior in rodents. The EPM consisted of two opposing open arms (30×8 cm) and two opposing closed arms (30×8×15 cm) that originated from a common central platform (8×8 cm), and are elevated 70 cm above the floor. At the start of the trial, animals were placed into an open field box for 5 min to avoid the mice hiding along the length of enclosed arms. Then, the mouse was placed in the center with the head facing towards an open arm and allowed to explore for 5 min. The percentage of open arms entries was calculated. All the data was recorded automatically using a video tracking system (SuperMaze software, Shanghai Xinruan Information Technology Co., Ltd, China).

Bioinformatics and Statistics for OMICs Data

RNA-Seq Data Analysis

We filtered and trimmed the reads using Trimmomatic v0.38 with parameters "HEADCROP:15 LEADING:20 TRAILING:20 SLIDINGWINDOW:5:20 MINLEN:50 AVGQUAL:20" [1]. Clean reads were mapped to the Mus musculus genome sequence (ftp://ftp.ncbi.nlm.nih.gov/genomes/all/GCF/000/001/635/GCF_000001635.26_GRCm38.p6) using Hisat2 v2-2.1.0 [2]. The reads per sample were then assembled into transcripts and compared with reference gene models using StringTie v1.3.4d (https://ccb.jhu.edu/software/stringtie/). StringTie emerges as a novel and widely used network flow algorithm aiming to assemble and quantitate full-length transcripts representing multiple splice variants for each gene locus [3]. We merged the 31 transcripts to obtain a consensus transcript using StringTie-Merge program. Transcripts that did not exist in the

CDS database of the Mus musculus genome were extracted to predicted new genes. The gene expression FPKM values were calculated using StringTie based on the consensus transcript. DEG analysis was performed using Ballgown v2.12.0 [4, 5, 6], which is an R programming based tool designed to facilitate flexible differential expression analysis of RNA-Seq data. We filtered the genes using ‘subset’ with parameters rowVars(gexpr(all_gene_fpkm))>1, and then obtained the differential expression genes using function ‘stattest’ with parameters false positive rate (FDR)-p < 0.05.

An unsupervised co-expression network analysis of all genes was performed using Weighted Correlation Network Analysis (WGCNA, R package WGCNA v1.64) [7]. Co-regulation networks describe functional relationships that can reflect both physical and non-physical interactions between objects including genes. The scale-free topology overlap matrix was computed using the ‘‘signed’’ and “bicor” parameter and using a best soft threshold power of 6 obtained from WGCNA function ‘pickSoftThreshold’, and co-expressing modules were then defined from this network. For each identified module of co-expression biomolecules, representative eigengenes were calculated (WGCNA function ‘moduleEigengenes’) and correlations between module eigengenes (ME) and phenotype data were calculated, as well as correlations between module eigengenes and each intra-module gene. For each identified module, the hub genes were defined by module connectivity (Pearson's correlation > 0.8) and correlations between each intra-module gene and treatments (correlation > 0.85). The co-expression network of hub genes was visualized using the free sofware Cytoscape [8]. The Gene ontology (and KEGG pathway were annotated using WebGestalt (http://www.webgestalt.org/2019/) and pathway with an false discovery rate (FDR) adjusted p-value of 0.05 considered to be significant.

16S rRNA Microbiome sequencing

The raw sequencing reads were merged and trimmed, following by removing chimera and constructing zero-radius Operational Taxonomic Units (zOTUs) with UNOISE implemented in Vsearch (v2.6.0) [9, 10, 11]. UNOISE is denoising algorithm to infer accurate biological template sequences from noisy illumina reads, which had comparable or better accuracy and much faster than DADA2 [12]. Raw reads were merged with fastq_mergepairs (Vsearch[2]) using defined parameters of fastq_minovlen = 16 and fastq_maxdiffs = 5. Merged reads were filtered with fastq_filter (Vsearch[2]) using defined parameters of fastq_truncqual = 4 and fastq_minleng = 400 and primers were chopped from both ends. In order to generate zOTUs, remaining high quality reads were dereplicated, clustering and denoised using derep_fullength, cluster_unoise and uchime3_denovo (Vsearch [2]) sequentially. Reads were mapped back to the zOTU sequence. The Greengenes (13.8) [13]. 16s rRNA gene database was used for taxonomy annotation of each zOTU using assign_taxonomy.py implemented in Qiime (v1.9.1) [14].

All the samples were rarefied to 28257 counts (lowest sample depth) to calculated the observed OTU index using alpha_diversity.py in Qiime (v1.9.1)[14]. Using normalized_table.py in Qiime (v1.9.1) [14]. The raw OTU table was normalized with cumulative sum scaling (CSS) [15] to calculate unweighted Unifrac distance. Permutational multivariate analysis of variance (PERMANOVA), a non-parametric multivariate statistical test, was adopted to detect differences among intervention groups using Adonis function in Vegan [16]. Constrained analysis of principal coordinate (CAP) in R package Vegan [16] was conducted to identify the influence of mice gene-type and IF on microbiota after setting time as a condition effect. The CSS normalized zOTU table was used to calculate relative abundance and summarized in different levels using Taxonomic-Binning. R in Rhea [17] Specific taxa

comparisons among groups was analyzed by analysis of composition of microbiomes (ANCOM) [18]. In brief, ANCOM algorithm,accounts for the underlying dependence structure of microbiota data, makes no distributional assumptions and scales well to compare samples involving thousands of taxa, which has been widely used in recent microbiota researches [18]. Using Correlation.R in Rhea [17], the Pearson correlation analysis was conducted between centered log-ratio transformed relative abundance of genera and body weight, blood glucose, food intake, water intake, lipopolysaccharide (LPS), leptin, *gamma*-Aminobutyric acid (GABA), 5-hydroxytryptamine (5-HT), insulin and short-chain-fatty-acids (SCFAs). The rarified OTU table was used to predict functional gene with Phylogenetic Investigation of Communities by Reconstruction of Unobserved States (PICRUSt v1.1.3) [19] following the official guide. PICRUSt helps to predict metagenome functional content from marker gene, including 16S rRNA surveys and full genomes. Briefly, the zOTU representative sequence were re-mapped with usearch_global in Usearch [20] to the reference OTU sequence in the Greengene (13.5) database as PICRUst utilized the same Greengenes 13.5 assigned OTUs to conduct the prediction. Then, the realigned zOTU table followed the standard PICRUst processing procedure using normalize_by_copy_number.py and predict_metagenomes.py. Predicted gene was annotated with KEGG [21] at different levels using categorize_by_function.py in PICRUst, and the significantly abundant pathways (at least appearing in 3 samples) were identified by edgeR [22] with FDR-p < 0.1.

Plasma metabolomics

The raw liquid chromatography-mass spectrometry (LC-MS) metabolomics data was processes using commercial software package Progenesis QI 2.0 (Nonlinear Dynamics; Newcastle upon Tyne, UK). Progenesis QI has emerged as a standard software for processing LC-MS metabolomics data and has been widely applied for data deconvolution,

peak-picking, alignment, and identifications of metabolites. Samples were analyzed in one batch with a randomized injection order. The stability and functionality of the system were monitored throughout all the instrumental analyses using quality controls, i.e. the pooling of all samples acquired at the beginning of analytical sequence and after every 10 injections. Data files of the information dependent acquisition scan mode were incorporated in the software for identification purposes to have MS/MS spectra of the most abundant detected metabolites. For the MS/MS detection, all precursors were fragmented using 20-40 eV, and the scan time was 0.2 seconds. During the acquisition, the signal was acquired every 3 seconds to calibrate the mass accuracy. Metabolite features that were detected in ≤ 50% QCs or 80% of biological samples were excluded. Missing values were imputed using k-nearest neighbor. Metabolite identification was carried out accurate mass (ppm<5) and product ion spectrum (MS/MS ppm<10) matching against different online databases including METLIN the Human Metabolome Database (HMDB, V4.0), NIST and Lipidblast. The list of microbial metablites (i.e. metabolites whose levels were modified by gut microbiota, n=26) was determined according to Rowan et al., 2017 [23] and detailed annotation information is provided in **Supplementary Spreadsheet 9**.

Integrated multi-omics analysis

Integrated multi-omics data analysis was performed on *a priori* selected parsimonious set of 36 genes, 17 ANCOM-derived OTUs that differed significantly between db/db and db/db-IF treatment and 26 pre-defined plasma microbial metablites. A detailed data processing workflow and R script are provided as an R markdown file.

First, multivariate predictive modelling on each omics dataset was conducted using partial least square-discriminant analysis incorporated into a repeated double cross-validation framework (rdCV-PLSDA) [24]. The rdCV separates cross validation into an outer “testing”

loop and an inner “tuning” (or validation) loop to effectively reduce bias from overfitting models to experimental data, which have shown better results than other cross-validation approaches [25] [26]. To gain a robust and reliable estimate of model performance, 200 repetitions of the outer cross validation loop was performed. Data was log-transformed and auto-scaled prior to the rdCV-PLSDA. We further applied permutation analysis (n=1000) to evaluate whether the constructed models outperformed than random classifications.

Second, a multivariate dimension reduction method, DIABLO (Data Integration Analysis for Biomarker discovery using a Latent component method for Omics), was employed for multiple omics integration [27]. DIABLO is a novel R programing based approach that is available in R package 'mixOmics', which is designd for objectively integrating multiple ‘omics datasets measured on the same biological samples. This algorithm is based on a variant of the multivariate methodology Generalised Canonical Correlation Analysis. Since each omics dataset has shown good predictive performance, as assessed by rdCV-PLSDA, we applied a full design matrix to seek for linear combinations of variables from each OMICs dataset that are maximally correlated (**Supplementary Figure 6A**). Subsequently, a tuning procedure (*tune.block.splsda* function) was applied to determine the optimal number of key predictors in each dataset for a minimum misclassification rate. Model performance was evaluated by 10-fold cross validation. The optimal number of component for each omics dataset was determined by rdCV-PLSDA. DIABLO model was then generated using *block.splsda.* A global overview of the correlation structure at the component level was represented with the plotDiablo function. A clustered image map that represents the multi-omics molecular signature expression for each sample was created using cimDiablo function. The loading weights of each selected variables on each component was represented with plotLoadings function.

Supplementary Table1 Key Resources in Current Study

Reagent or resource	Source	Identifier
Antibodies		
Claudin-1	Abcam	Cat# ab15098; RRID:AB_301644
Iba-1	Abcam	Cat# ab178847
mTOR	Abcam	Cat# ab2732; RRID:AB_303257
Anti-mTOR (phospho S2448)	Abcam	Cat# ab109268; RRID:AB_10888105
PPARα	Abcam	Cat# ab8934; RRID:AB_306869
Phospho-IRS-1 (Tyr896)	Abcam	Cat# ab46800; RRID:AB_881460
AKT	Cell Signaling Technology	Cat# cs9272; RRID:AB_329827
AMPKα	Cell Signaling Technology	Cat# cs2603; RRID:AB_490795
CREB	Cell Signaling Technology	Cat# cs9197; RRID:AB_331277
p44/42 MAPK (Erk1/2)	Cell Signaling Technology	Cat# cs9102; RRID:AB_330744
SAPK/JNK	Cell Signaling Technology	Cat# cs9252; RRID:AB_2250373
NF-κB p65	Cell Signaling Technology	Cat# cs8242; RRID:AB_10859369
Phospho-SAPK/JNK (Thr183/Tyr185)	Cell Signaling Technology	Cat# cs9255; RRID:AB_2307321
p38 MAPK	Cell Signaling Technology	Cat# cs8690; RRID:AB_10999090
Phospho-Akt (Ser473)	Cell Signaling Technology	Cat# cs4060; RRID:AB_2315049
Phospho-AMPKα (Thr172)	Cell Signaling Technology	Cat# cs2535; RRID:AB_331250
Phospho-CREB (Ser133)	Cell Signaling Technology	Cat# cs9198; RRID:AB_2561044
Phospho-NF-κB p65 (Ser536)	Cell Signaling Technology	Cat# cs3033; RRID:AB_331284
Phospho-p38 MAPK (Thr180/Tyr182)	Cell Signaling Technology	Cat# cs4511; RRID:AB_2139682
Phospho-p44/42 MAPK (Erk1/2) (Thr202/Tyr204)	Cell Signaling Technology	Cat# cs9101; RRID:AB_331646
PSD-95	Cell Signaling Technology	Cat# cs3450; RRID:AB_2292883
BDNF	SANTA CRUZ	Cat# sc65514; RRID:AB_1128219
COX4	SANTA CRUZ	Cat# sc69360; RRID:AB_2085281
IRS-1	SANTA CRUZ	Cat# sc559; RRID:AB_631842
ND1	SANTA CRUZ	Cat# sc20493; RRID:AB_2149734
PGC-1α	SANTA CRUZ	Cat# sc518025
α-tubulin	SANTA CRUZ	Cat# sc5286; RRID:AB_628411

β-actin	SANTA CRUZ	Cat# sc1616; RRID:AB_630836
Goat anti-Rabbit IgG (H+L) Cross-Adsorbed Secondary Antibody, HRP	Thermo Fisher Scientific	Cat# a16110; RRID:AB_2534783
Goat anti-Mouse IgG (H+L) Cross-Adsorbed Secondary Antibody, HRP	Thermo Fisher Scientific	Cat# a16078; RRID:AB_2534752
HRP-conjugated Affinipure Rabbit Anti-Goat IgG(H+L)	Proteintech	Cat# sa00001-4
Primers		
APLP2	ACCTGGAGCAGATGCAGATT	TCATGCACAACCCAGAACAT
Atp5d	GCTGAAGAAGCTGTGACACT	TTGGCCTCAATACGGATCTG
b-globin	GAAGCGATTCTAGGGAGCAG	GGAGCAGC GATTCTGAGTAGA
Claudin-1	CGACTCCAAACACTGGAACTCA	GCCTGCTTCTCATCTGTTGTCA
COX2	GCCGACTAAATCAAGCAACA	CAATGGGCATAAAGCTATGG
Gpx4	TACCGGGGCTTCGTGTGCAT	TAGCCCGCGGCGAACTCTTT
ND4	CCATTCTCCTCCTATCCCTCAAC	CACAATCTGATGTTTTGGTTAAACT ATATTT
NDUFA	TTATGGGGGTGTGCTTGGTC	GTTTTTCCTTGCCCCCGTTG
NT5C	CGCTTTGCGAGAGATGAACG	GTCTCCTCTAGGCCTTGAATGT
SOD	CGGATGAAGAGAGGCATGTT	GTACGGCCAATGATGGAATG
Uqcrc1	TGCTGAGCAGGTTTCTAGGC	TCCTTCTTAAACTTGCCGTTG
16S	AGAGTTTGATCCTGGCTCAG	CTGCTGCCTCCCGTAGGAGT
PSD95	TCTGTGCGAGAGGTAGCAGA	AAGCACTCCGTGAACTCCTG
GADPH	TGGAGAAACCTGCCAAGTATGA	TGGAAGAATGGGAGTTGCTGT
Chemicals		
Metronidazole	Dalian Meilun biological Technology Co	CAS 443-48-1
Penicillin G sodium	Dalian Meilun biological Technology Co	CAS 69-57-8
Streptomycin sulfate	DIYIbio	CAS 3810-74-0
Vancomycin hydrochloride	DIYIbio	CAS 1404-93-9
Neomycin Sulfate	MP biomedicals	CAS 100541
Hematoxylin	Poly-scientific	Cat# S212
Eosin	StatLab	Cat# SL98-1；CAS:2321-07-5
Insulin	Solarbio	Cat# I8040；CAS:11070-73-8

TB Green™ Premix Ex Taq™ II	TaKaRa	Cat# RR820Q
Sodium dodecyl sulfate	MP biomedicals	Cat# 190522
Fluorescein - Reference Standard	Thermo Fisher Scientific	Cat# F1300
Acetic acid	Aladdin	Cat# A116173
Propionic acid	Aladdin	Cat# P110446
Butyric acid	Aladdin	Cat# B110438
Experimental Models: Organisms		
Mouse: BKS.Cg-Dock7m+/+ Leprdbdb/J	The Jackson Laboratory	Stock No: 000642
Sterile chow diet	TROPHIC Animal Feed High-tech Co.	AIN-93M
Critical Commercial Assays		
TRIzol Kit	Jingcai Bio.	JC-PR001
Stool DNA Kit	Omega	D4015
DNA extraction Kit	Bioteke Co.	DP1901
Leptin Kit	Xinle Bio.	xl-Em0230
Insulin Kit	Xinle Bio.	xl-Em0483
5-HT Kit	Xinle Bio.	xl-Em1037
TC Kit	Nanjing Jiancheng Bio.	A111-1
TG Kit	Nanjing Jiancheng Bio.	A110-1
HDL Kit	Nanjing Jiancheng Bio.	A112-1
LDL Kit	Nanjing Jiancheng Bio.	A113-1
LPS Kit	Xinle Bio.	xl-Em1110
Software and Algorithms		
ImageJ v1.42	National Institutes of Health	RRID:SCR_003070
GraphPad Prism 7	GraphPad Software	https://www.graphpad.com/scientificsoftware/prism/
R version 3.5.1	R Core Team, 2018	https://www.r-project.org/
R package MixOmics	[28]	http://mixomics.org/
R package MetNormalizer	[29]	https://link.springer.com/article/10.1007/s11306-016-1026-5

R package WGCNA	[7]	https://bmcbioinformatics.biomedcentral.com/articles/10.1186/1471-2105-9-559
R package MUVR	[24]	https://academic.oup.com/bioinformatics/advance-article/doi/10.1093/bioinformatics/bty710/5085367
Progenesis QI (version 2.2)	Waters company	http://www.nonlinear.com/progenesis/qi/
Qiime (version 1.9.1)	[30]	http://qiime.org/
Vsearch (version 2.6.0)	[10],[31]	https://github.com/torognes/vsearch
R package ANCOM	[18]	https://www.niehs.nih.gov/research/resources/software/biostatistics/ancom/index.cfm
R package Rhea	[17]	https://github.com/Lagkouvardos/Rhea
PICRUst (version 1.1.3)	[19]	http://picrust.github.io/picrust/
R package WGCNA (version 1.64)	[7]	https://horvath.genetics.ucla.edu/html/CoexpressionNetwork/Rpackages/WGCNA/index.html
WebGestalt	http://www.webgestalt.org	http://www.webgestalt.org/2019/
Deposited Data		
Raw and processed data (RNA-seq)	GEO (http://www.ncbi.nlm.nih.gov/geo/)	GSE125387<br>The secure token for reviewer is: opwxoiucndybjor
Other raw data	Mendeley dataset	https://figshare.com/s/58ccac4aa614dd4ade84

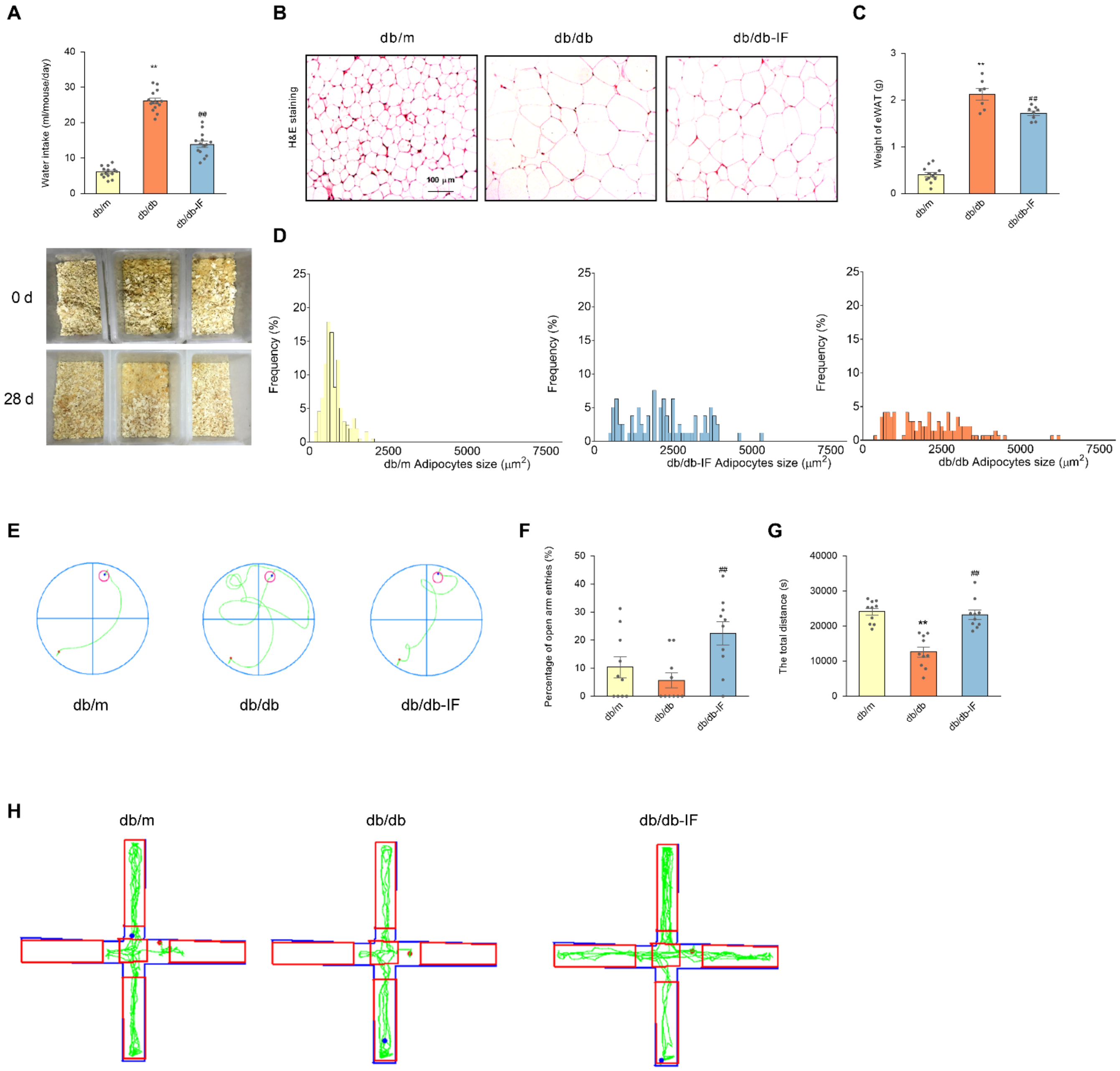

Supplementary Figure 1 Effects of IF on adipocytes size, cognition, and anxiety in diabetic mice, related to Fig.2, related to Fig.1

A Water intake (n=10 mice per group) and cage padding of each group; **B** H&E staining of eWAT (n=3, 4, 3 mice in db/m, db/db, db/db-IF group, respectively); **C** eWAT weight (n=7 mice per group); **D** Distribution of adipocytes in eWAT (n=3, 4, 3 mice in db/m, db/db, db/db-IF group, respectively); **E** The representative tracks of mice on the 5th day of navigation test during the water maze test; The assessments of depression via the

Elevated plus maze tests were described in the Supplementary Methods; **F** Total distance (n=10 mice per group) and **G** percentage of the mice spent in the open arm entries compared with the total ones (n=10 mice per group); **H** Representative tracks of mice in elevated plus maze. Data presented as mean ± SEM. *$p < 0.05$, **$p < 0.01$, compared with db/m group, $^{\#}p < 0.05$, $^{\#\#}p < 0.01$ versus db/db group. Significant differences between mean values were determined by one-way ANOVA with Tukey's multiple comparisons test. Source data are provided as a Source Data file.

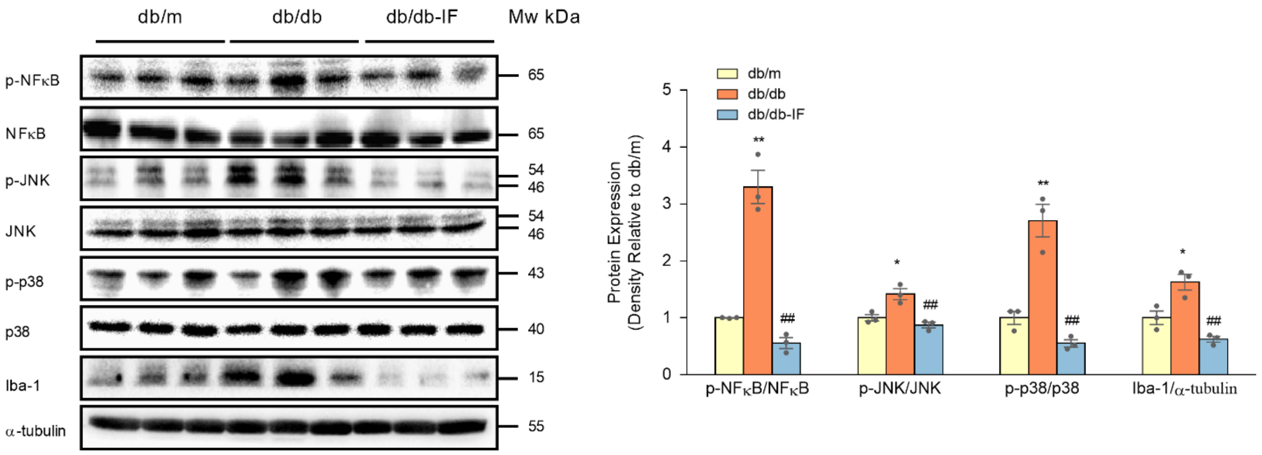

Supplementary Figure 2 Effects of IF on neuroinflammation-related signaling in diabetic mice, related to Fig.2

Western blots of NFκB/JNK/p38/Iba-1 signaling (n=3 mice per group). Data presented as mean ± SEM. *p < 0.05, **p < 0.01, compared with db/m group, #p < 0.05, ##p < 0.01 versus db/db group. Significant differences between mean values were determined by one-way ANOVA with Tukey’s multiple comparisons test. Source data are provided as a Source Data file.

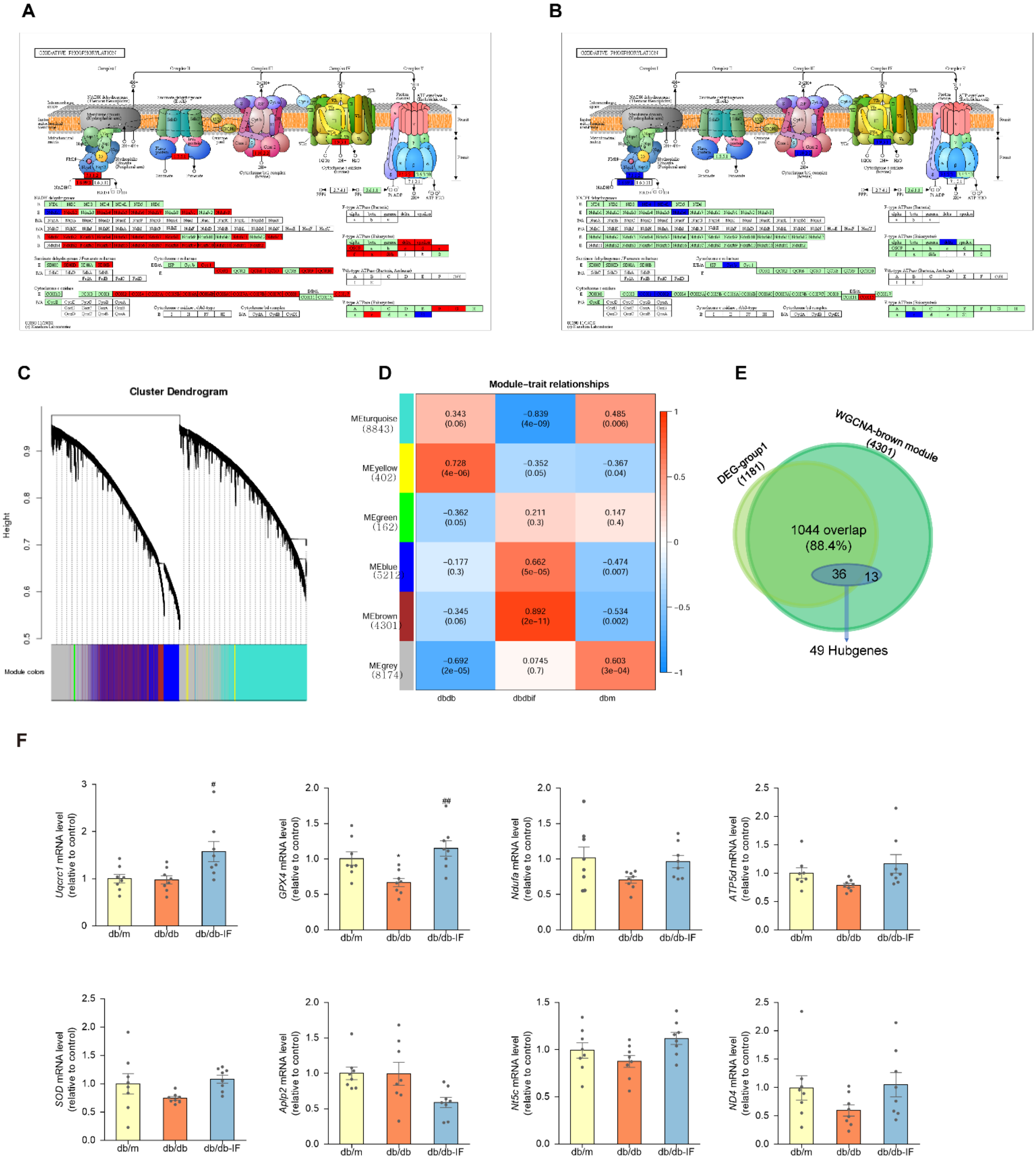

Supplementary Figure 3 The KEGG analysis of DEG and the modules of WGCNA, related to Fig.3

A KEGG of DEG analysis (group 1 oxidative phosphorylation); **B** KEGG of DEG analysis (group 3 & group 6 oxidative phosphorylation); **C** Hierarchical cluster tree showing 5 modules of co-expressed genes (TOMType ="signed", corType = "bicor"). Each of the 27,094 genes is represented by a tree leaf and each of the modules by a major tree branch. The lower panel shows modules in designated colours; **D** Module–trait correlations and corresponding p-values (in parentheses). The left panel shows the 5 modules and the number of member genes. The colour scale on the right shows module–trait correlations from -1 (blue) to 1 (red); **E** Overlap genes numbers of group 1 DEG analysis and WGCNA-brown module; **F** qPCR analysis of mitochondrial biogenesis and specific genes from DEG and WGCNA analysis (n=8 biologically independent samples per group). Data of **F** presented as mean ± SEM; **G** $^{*}p < 0.05$, $^{**}p < 0.01$, compared with db/m group, $^{\#}p < 0.05$, $^{\#\#}p < 0.01$ versus db/db group. Significant differences between mean values were determined by one-way ANOVA with Tukey's multiple comparisons test. Source data are provided as a Source Data file.

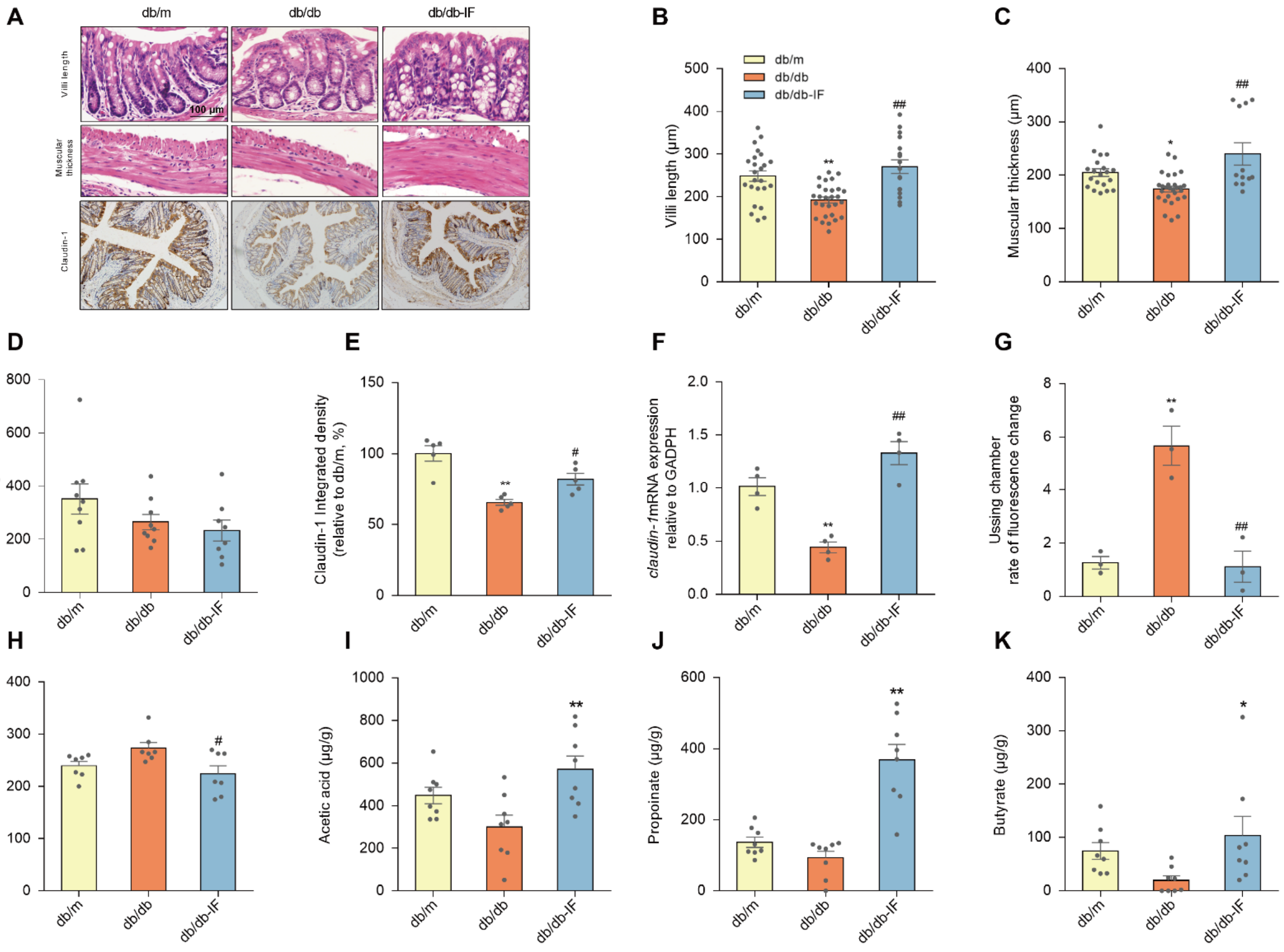

Supplementary Figure 4 Effects of IF on gut barrier permeability and SCFAs generation in db/db mice related to Fig.4

A Representative images and analysis of H&E staining of the colon (n=9, 9, 8 mice in db/m, db/db, db/db-IF group, respectively) and the immunochemical staining of claudin-1 (n = 5 mice per group), based on **B** The villi length (n=9 mice per group); **C** The muscular thickness; **D** The numbers of goblet cell; and **E** the claudin-1 integrity; **F** qPCR analysis of claudin-1 (n=4 mice per group); **G** The rate of fluorescence changes during the using chamber analysis (n=3 mice per group); **H** Plasma LPS level (n=7 mice per group); **I-K** the concentrations of short chain fatty acids in the fecal samples (n=8 mice per group). Data presented as mean ± SEM. *p < 0.05, **p < 0.01, compared with db/m group, #p < 0.05, ##p < 0.01 versus db/db group. Significant differences between mean values were determined by one-way ANOVA with Tukey's multiple comparisons test. Source data are provided as a Source Data file.

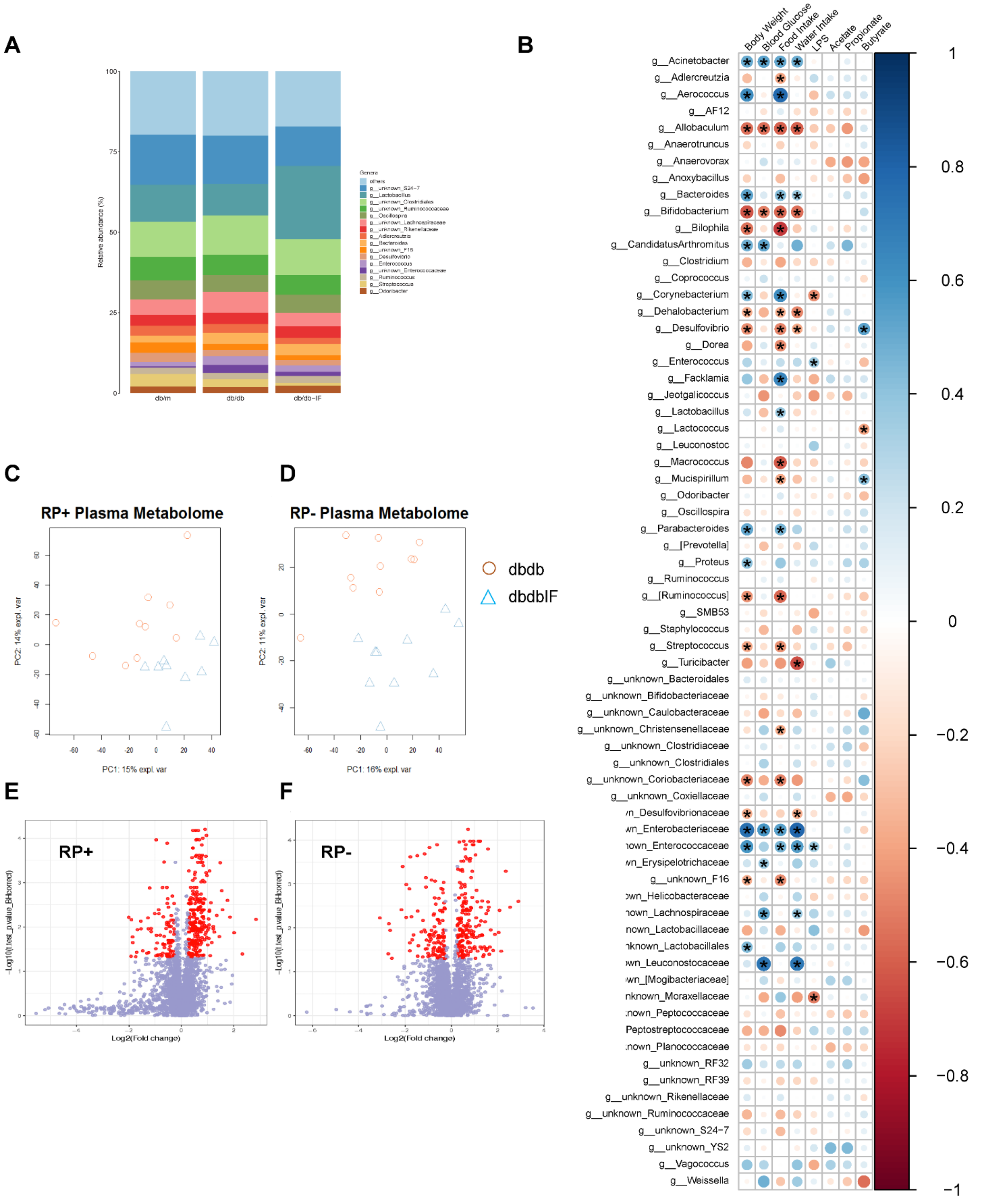

Supplementary Figure 5 Effects of IF on gut microbiome and plasma metabolome in db/db mice related to Fig.4

A The relative abundance of prokaryotic Microbiota members at genus level, all genera with an average relative abundance below 1% were grouped to “others”; **B** Pearson correlationsbetween genera relative abundance (centered log-ratio transformed) and body weight, blood glucose, food intake, water intake, LPS, and SCFAs; **C** The scatter plots of principal component analysis performed on mice plasma untargeted metabolomics data aqucised by peverse phase chromatography using both positive (**C**, RP+) and negative electrospray ionization mode (**D**, RP-). Principal component analysis was performed on auto-scaled intensities (mean=0, standard deviation=1) of all quantified metabolite features detected in RP+ (5604) and RP- (5230), respectively. Volcano plots of qualified features from RP+ (**E**) and RP- (**F**) shows fold change in plasma levels of metabolite features between db/db and db/db-IF and their significances. p-values were adjusted for multiple testing using Benjamini–Hochberg false discovery rate (FDR). Significant features are marked in red.

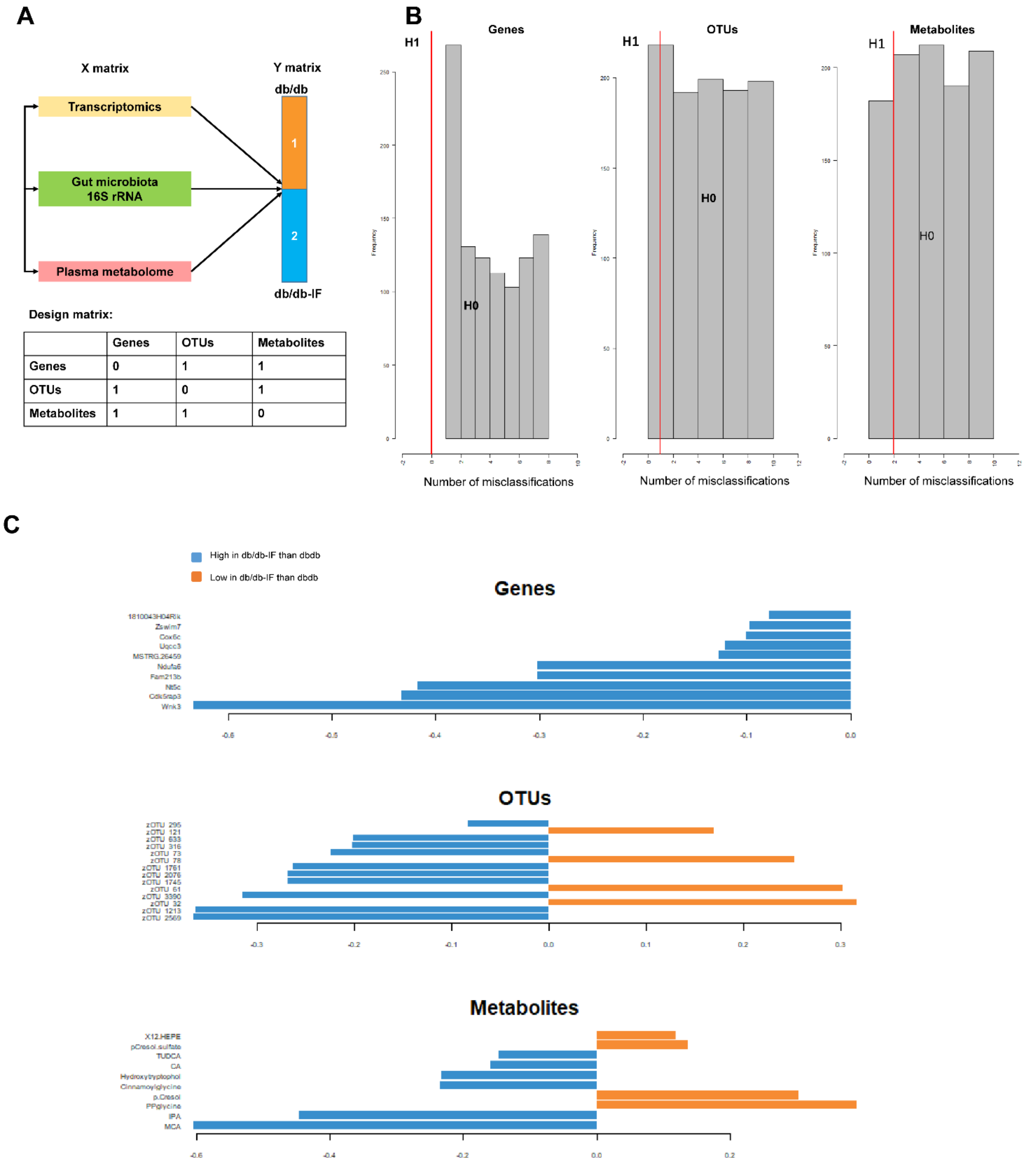

Supplementary Figure 6 Predictive performance of variables from multi-omics datasets that were selected as input variables for DIABLO and their variable loadings obtained from DIABLO, related to Fig.5

A Graphic illustration of the full design matrix in DIABLO used for the multi-omics integration analysis; **B** Permutation analysis of multivariate predictive modelling. H1 denotes number of misclassification obtained

from actual models. H0 are distributions of misclassification from random permutations. Model performance of variables from multi-OMICs datasets outperformed random permutations (One-tailed Student's t-test, $p < 0.05$). Variables loading reflected the contribution of variables for DIABLO modeling; **C** Pyramid barplot displays the loading weights associated to each selected feature in increasing order of importance (from bottom to top). The loading plot represents the top 10 contributors selected from each of omics datasets on the first component of the DIABLO model. Colors indicate the sample group, i.e. db/db and db/db-IF where the mean expression levels of the variables is maximal.

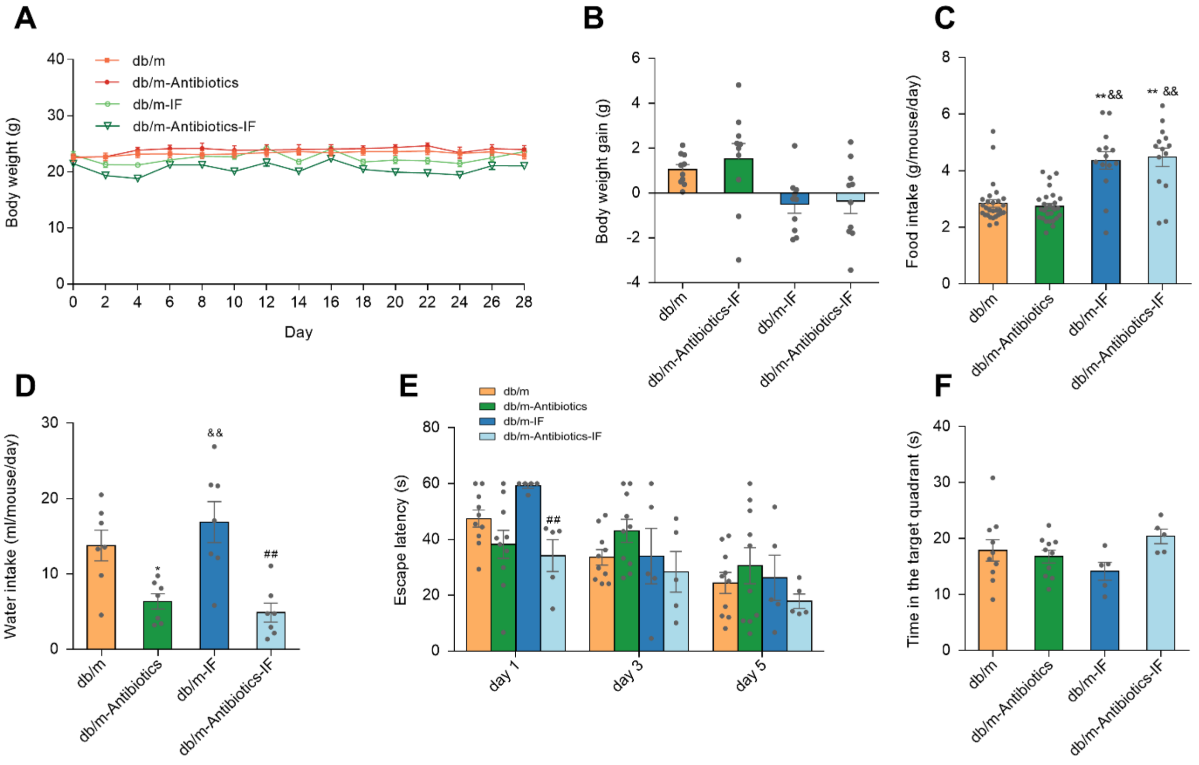

Supplementary Figure 7 Effects of antibiotics treatment and IF regimen on db/m mice, related to Fig.6

The mice were administrated with antibiotics in the drinking water starting 14 days before the 4-week IF regimen and throughout the experiment (The detailed antibiotics treatment was as described in Methods section) (n=10 mice per group); **A** Body weight; **B** Bodyweight gain; **C** Food intake; **D** Water intake; **E-F** Morris water maze test (n=10, 10 ,5, 5 mice in db/m, db/m-Antibiotics, db/m-IF and db/m-Antibiotics-IF group, respectively). Data presented as mean ± SEM. *p < 0.05, **p < 0.01, compared with db/m group, $^{\&}p < 0.05$, $^{\&\&}p < 0.01$, compared with db/dm-Antibiotics group, $^{\#}p < 0.05$, $^{\#\#}p < 0.01$ versus db/dm-IF group. Significant differences between mean values were determined by two-way ANOVA (IF regimen and antibiotics treatment as two factors) with Tukey's multiple comparisons test. Source data are provided as a Source Data file.

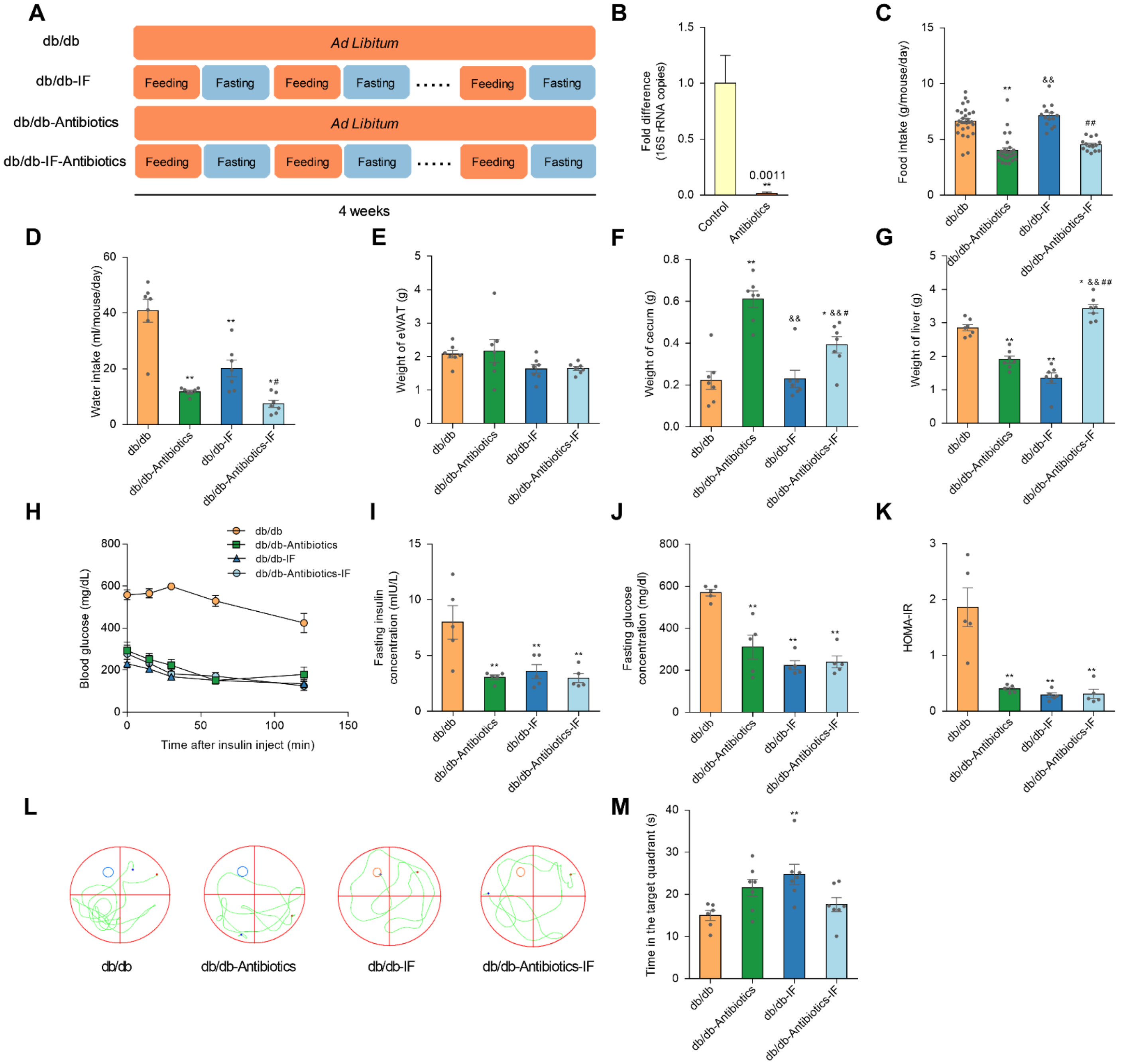

Supplementary Figure 8 Effects of antibiotics treatment and IF regimen on db/db mice, related to Fig.6

The mice were administrated with antibiotics in the drinking water starting 14 days before the 4-week IF regimen and throughout the experiment (The detailed antibiotics treatment was as described in Methods section). **A** Timeline depicting the treatment of IF and antibiotics on db/db mice (n=7, 13 mice in non-antibiotics and antibiotics treatment groups, respectively); **B** The qPCR of 16S rRNA analysis to ensure the removal

efficacy of microbiota, as assessed byStudent's t-test, ** $p < 0.01$, compared with control mice fed with normal water; **C** Food intake; **D** Water intake; **E-G** The weight of eWAT, liver, and cecum in different groups (n=7 mice per group); **H** Insulin tolerance test (n=7 mice per group); **I** Fasting insulin level (n=5 mice per group); **J** Fasting glucose (n=5 mice per group); **K** HOMA-IR value (n=5 mice per group); **L** The representative tracks of mice on the 5th day of navigation test and **M** the time spent in the target quadrant (s) during the probe trial during the water maze test (n=7 mice per group). Data presented as mean ± SEM. *$p < 0.05$, **$p < 0.01$, compared with db/db group, $^{\&}p < 0.05$, $^{\&\&}p < 0.01$, compared with db/db- Antibiotics group, $^{\#}p < 0.05$, $^{\#\#}p < 0.01$ compared with db/db-IF group. Significant differences between mean values were determined by two-way ANOVA (IF regimen and antibiotics treatment as two factors) with Tukey's multiple comparisons test. Source data are provided as a Source Data file.

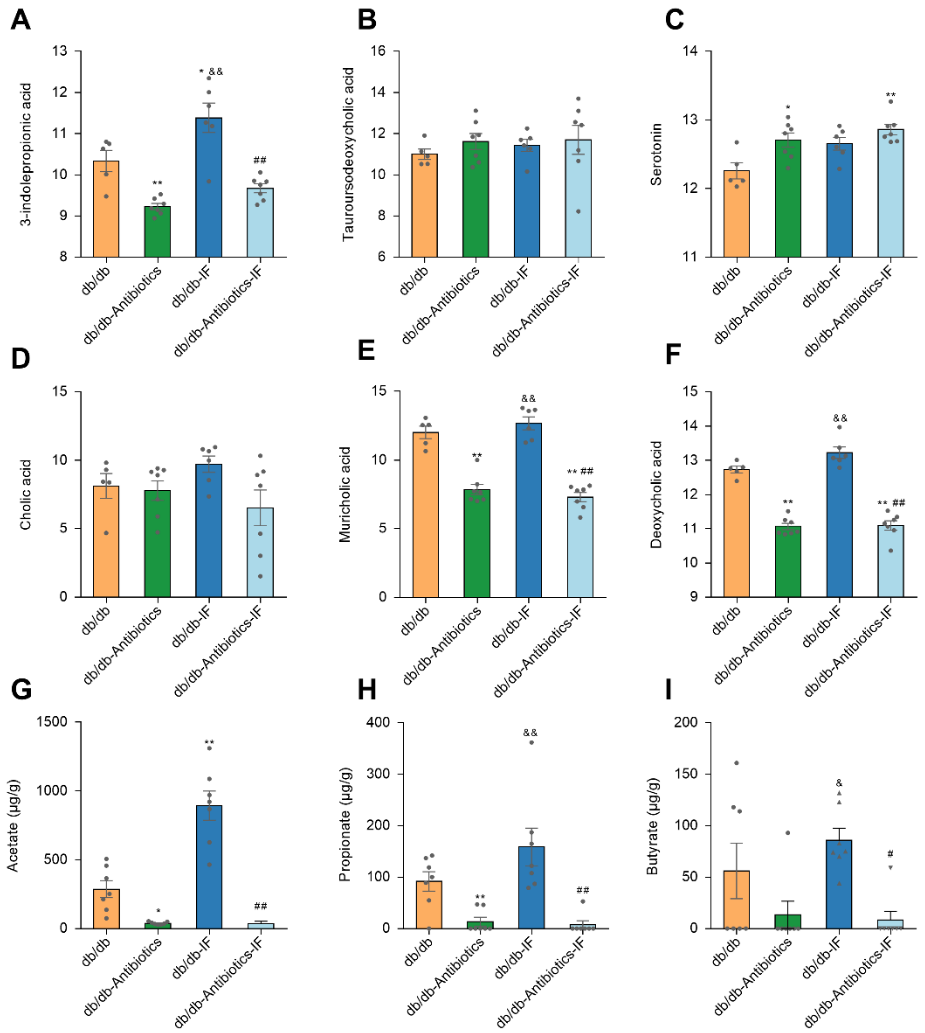

Supplementary Figure 9 Effects of antibiotics treatment and IF regimen on the microbial metabolites levels in db/db mice, related to Fig.6

A-F The plasmamicrobial metabolites alteration of antibiotics-treated mice (n=5, 7, 6, 7 mice in different group) detected by metabolome. The unit is the log10 value of relative abundance; **G-I** The alteration of fecal SCFAs levels of of antibiotics-treated mice (n=7 mice per group). Data presented as mean ± SEM. $^{*}p < 0.05$, $^{**}p < 0.01$, compared with db/m group, $^{\&}p < 0.05$, $^{\&\&}p < 0.01$, compared with db/dm-Antibiotics group, $^{\#}p < 0.05$, $^{\#\#}p < 0.01$ versus db/m-IF group. Significant differences between mean values were determined by two-way ANOVA (IF regimen and antibiotics treatment as two factors) with Tukey's multiple comparisons test. Source data are provided as a Source Data file.

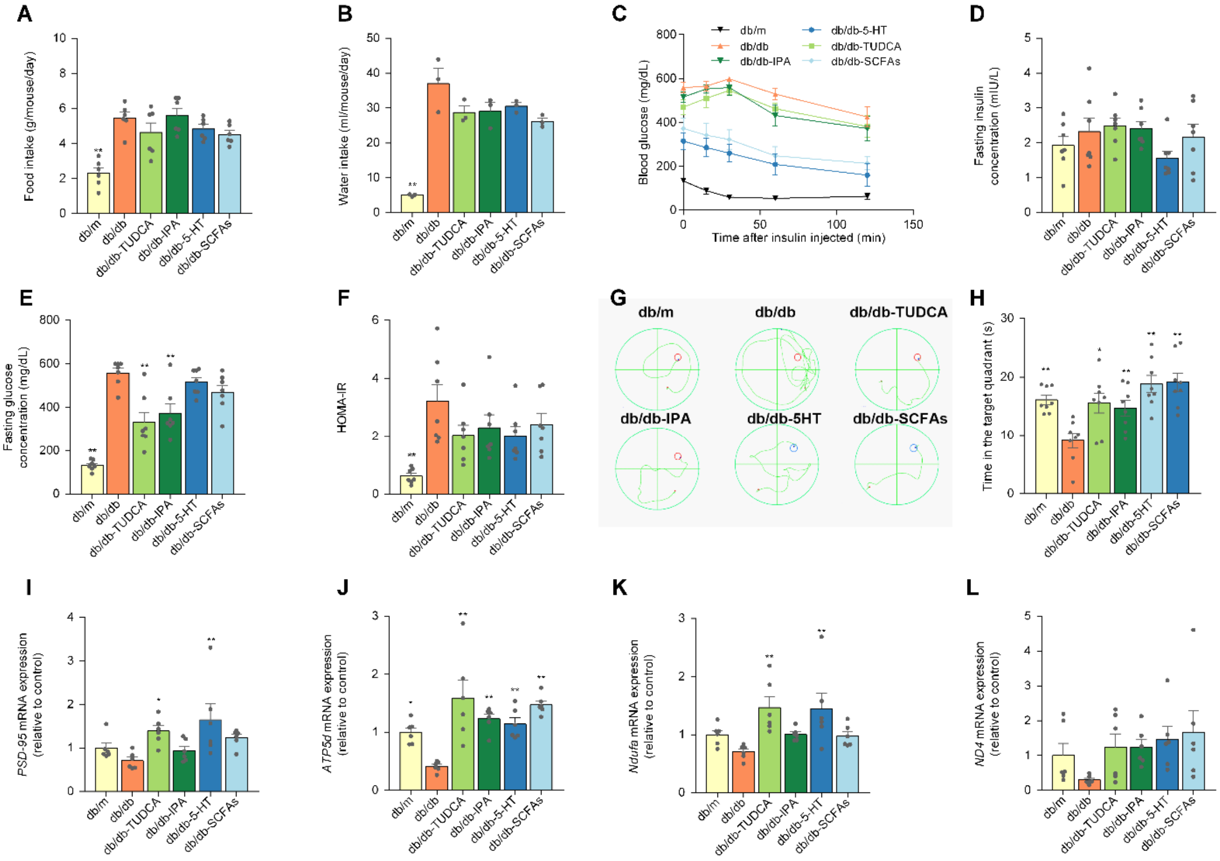

Supplementary Figure 10 Effects of selected microbial metabolites on db/db mice, related to Fig.6

The db/db mice were administratedwith IPA, 5-HT, TUDCA, or SCFAs, i.e acetate, butyrate and propionate, individually (n=8 mice per group). The treatment was described in the Methods section. **A** Food intake; **B** Water intake; **C** Insulin tolerance test (n=7 mice per group); **D** Fasting insulin level (n=7 mice per group); **E** Fasting glucose (n=7 mice per group); **F** HOMA-IR value (n=7 mice per group); **G** The representative tracks of mice on the 5th day of navigation test and **H** the time spent in the target quadrant (s) during the probe trial during the water maze test (n=8 mice per group); **I-L** qPCR analysis of mitochondrial biogenesis and specific genes from DEG and WGCNA analysis (n=6 mice per group); Data presented as mean ± SEM. *p < 0.05, **p < 0.01 versus db/db group. Significant differences between mean values were determined by one-way ANOVA with Tukey's multiple comparisons test. Source data are provided as a Source Data file.

R markdown file for multi-omics analysis

This is an R Markdown document that presents the detailed procedure for multi-omics analysis.

In brief, we first conducted multivariate predictive modellings on WGCNA-derived hub genes (n=36), ANCOM-derived OTUs (n=17) and the predefined microbial metabolites using partial least square-discriminant analysis incorporated into a repeated double cross-validation framework (rdCV-PLSDA). Outperforming the standard cross-validation, the double cross-validation procedure separates cross-validation into an outer “testing” loop and an inner “tuning” (or validation) loop to further reduce bias from overfitting models to experimental data. To gain a robust and reliable estimate of model performance, 200 repetitions of the outer cross-validation loop was performed, followed by permutation analysis (n=1000).

A multivariate dimension reduction method, DIABLO (Data Integration Analysis for Biomarker discovery using a Latent component method for Omics), was employed for multiple omics integration. A use of full design matrix was applied to seek for linear combinations of variables from each omics dataset that are maximally correlated. The specified number of components for each omics dataset is determined by rdCV-PLS. A tuning procedure was applied to determine the optimal number of key variables in each dataset to be selected with a minimum misclassification rate and the model performance is then evaluated by 10-fold cross validation.

Description of datasets

Gene: WGCNA-derived hub genes (n=36)

OTU: ANCOM-derived OTUs (n=17)

Metabolite: a priori defined microbial metabolites (n=27)

Integrated Multi-omics Analysis Procedure

- **Step 1: Load packages for analyses and check data information**

```
## Loading required package: foreach

## Loading required package: iterators

## Loading required package: parallel

##  [1] "dbdb"   "dbdb"   "dbdb"   "dbdb"   "dbdb"   "dbdb"   "dbdb"
##  [8] "dbdb"   "dbdb"   "dbdb"   "dbdbIF" "dbdbIF" "dbdbIF" "dbdbIF"
## [15] "dbdbIF" "dbdbIF" "dbdbIF" "dbdbIF" "dbdbIF" "dbdbIF"

##           Tmem160    Xrcc1    Psmc4  Ndufa13      Gpx4
## db_db_1  2.669484 19.26067 112.6700 19.95793 105.79840
## db_db_10 4.036271 20.44588 123.8790 27.96805 144.66346
## db_db_2  2.611742 21.17438 115.4641 20.69619  98.19304
## db_db_3  2.648136 19.85958 114.0726 20.81813 105.87250
## db_db_4  3.327520 21.75117 120.6406 24.52895 120.38551
## db_db_5  3.528933 18.48286 117.9990 23.22875 128.52778

##          zOTU_105 zOTU_111 zOTU_121 zOTU_1213 zOTU_122
## db_db_1    8.4187   8.1318   4.9934         0   6.6556
## db_db_10   7.9026   8.0976   0.0000         0   7.5730
## db_db_2    4.9509   4.9509   2.6512         0   5.8414
## db_db_3    5.4095   4.7383  11.9120         0   5.9603
## db_db_4    6.0469   7.8090   2.4985         0   3.9024
## db_db_5    8.6686   6.8317   6.6505         0   7.2435

##           Acetate Butyrate Propionate      TCDA Indole.3.pyruvate
## db_db_1  489.2959 277.4953   89.53000 148474.29          52986.66
## db_db_10 312.4716 121.4445   62.33240 110637.89          42107.37
## db_db_2  361.4286 134.0848   59.57483 170988.32          70187.62
## db_db_3   51.8564 150.7360   69.12925 108931.42          59676.15
## db_db_4  891.5903 303.9216  113.28207  80591.94          42731.24
## db_db_5  532.9761 130.7090   23.06470 114313.00          68074.64
```

- **Step 2: Perform comprehensive cross-validated partial least square-discriminant analysis (rdCV-PLSDA) on each omics dataset to assess their predictive performance** It may take 3-5 mins for processing, due to multiple cross validations.

```
set.seed(123)#### for reproducibility
cl=makeCluster(2)
registerDoParallel(cl)
Group=OMICsD$Group
GeneD=scale(log(OMICsD$Gene+0.01),center=T,scale=T)###36b overlapped genes
G_mod=rdCV(X=GeneD,Y=Group,nRep=200,method='PLS',fitness = 'MISS')

## Type 'citation("pROC")' for a citation.

##
## Attaching package: 'pROC'
```

```
## The following objects are masked from 'package:stats':
##
##     cov, smooth, var

##
## Missing ID -> Assume all unique (i.e. sample independence)
## Y is factor -> Classification (2 classes)
##  Elapsed time 0.6753333 mins

OTUD=scale(log(OMICsD$OTU+0.01),center=T,scale=T)
O_mod=rdCV(X=OTUD,Y=Group,nRep=200,method='PLS',fitness = 'MISS')

##
## Missing ID -> Assume all unique (i.e. sample independence)
## Y is factor -> Classification (2 classes)
##  Elapsed time 0.8393333 mins

MetaboliteD=scale(log(OMICsD$Metabolite+0.01),center=T,scale=T)
M_mod=rdCV(X=MetaboliteD,Y=Group,nRep=200,method='PLS',fitness = 'MISS')

##
## Missing ID -> Assume all unique (i.e. sample independence)
## Y is factor -> Classification (2 classes)
##  Elapsed time 0.6541667 mins

par(mfrow = c(1, 3), pty = "m",mar=c(3,4,2,1), par(cex.lab=1,las=2) )
plotMV(G_mod)
plotMV(O_mod)
plotMV(M_mod)
```

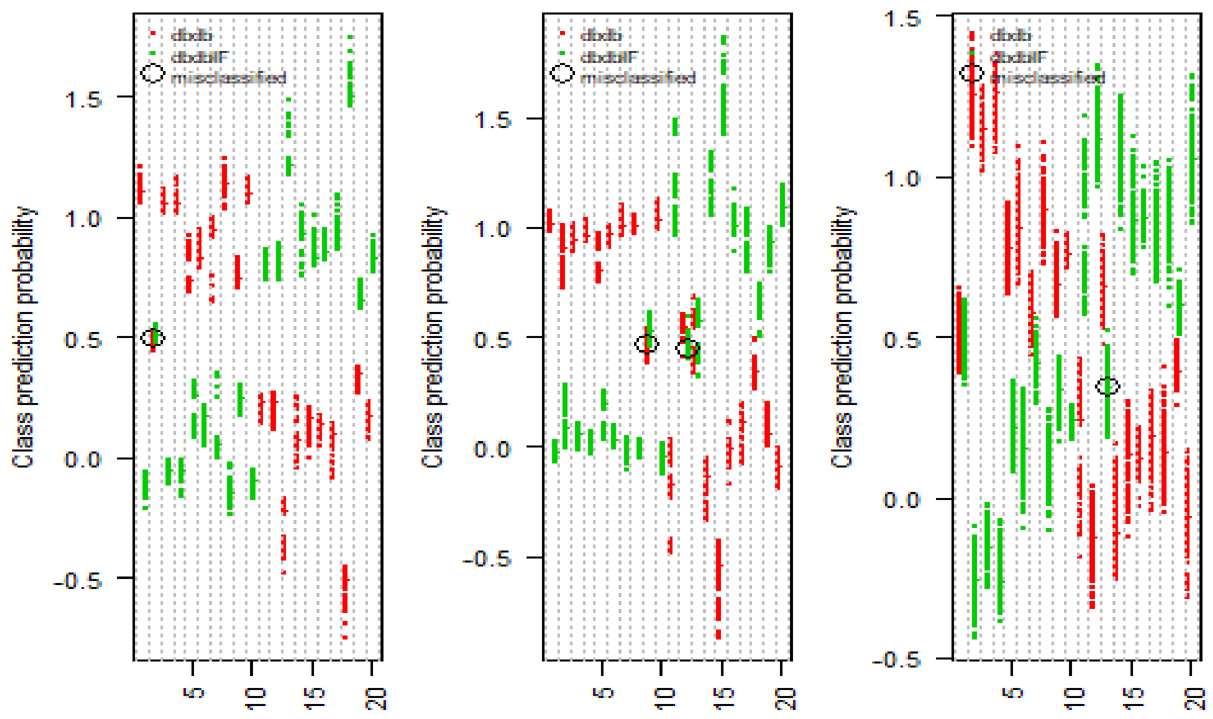

- **Step 3: Perform DIABLO for integrative modelling of genes, OTUs and metabolites**

```
library(mixOmics)

## Loading required package: MASS

## Loading required package: lattice

## Loading required package: ggplot2

##
## Loaded mixOmics 6.6.2
##
## Thank you for using mixOmics! Learn how to apply our methods with our tutorials on www.mixO
mics.org, vignette and bookdown on  https://github.com/mixOmicsTeam/mixOmics
## Questions: email us at mixomics[at]math.univ-toulouse.fr
## Bugs, Issues? https://github.com/mixOmicsTeam/mixOmics/issues
## Cite us:  citation('mixOmics')

##
## Attaching package: 'mixOmics'

## The following objects are masked from 'package:rdCV':
##
##     nearZeroVar, pls, plsda, vip

data = list(Genes = GeneD,OTUs = OTUD,Metabolites =MetaboliteD)### set dataset
rownames(data$Genes)=rownames(data$OTUs)=rownames(data$Metabolites)=OMICsD$ID##
# set rownames for each dataset
lapply(data, dim)## check datasets

## $Genes
## [1] 20 36
##
## $OTUs
## [1] 20 17
##
## $Metabolites
## [1] 20 26

Y = as.factor(c(rep('dbdb',10),rep('dbdbIF',10)))
design = matrix(1, ncol = length(data), nrow = length(data),dimnames = list(names(data), name
s(data)))
##A full design matrix was applied to seek for linear combinations of variables from each omics dat
aset that are maximally correlated.
diag(design) = 0
ncomp=G_mod$nComp=O_mod$nComp=M_mod$nComp### the number of component is determ
ined by the rdCV-PLS
test.keepX = list(OTUs =c(seq(10, 17, 4)), Genes = c(seq(10, 36, 4)), Metabolites = c(seq(10, 26,
 4)))
tune = tune.block.splsda(X = data, Y = Y, ncomp = ncomp, test.keepX = test.keepX,
      design = design,validation = 'Mfold', folds = 10, nrepeat = 20,cpus = 2)
```

```
## You have provided a sequence of keepX of length: 2 for block OTUs and 7 for block Genes and
 5 for block Metabolites.
## This results in 70 models being fitted for each component and each nrepeat, this may take som
e time to run, be patient!

## As code is running in parallel, the progressBar will only show 100% upon completion of each nr
epeat/ component.

##
## comp 1
##
  |
  |                                                                      |   0%
  |
  |===                                                                   |   5%
  |
  |=======                                                               |  10%
  |
  |==========                                                            |  15%
  |
  |==============                                                        |  20%
  |
  |==================                                                    |  25%
  |
  |=====================                                                 |  30%
  |
  |=========================                                             |  35%
  |
  |============================                                          |  40%
  |
  |================================                                      |  45%
  |
  |===================================                                   |  50%
  |
  |======================================                                |  55%
  |
  |==========================================                            |  60%
  |
  |=============================================                         |  65%
  |
  |=================================================                     |  70%
  |
  |====================================================                  |  75%
  |
  |========================================================              |  80%
  |
  |============================================================          |  85%
  |
  |===============================================================       |  90%
  |
  |==================================================================    |  95%
  |
  |======================================================================| 100%
```

Here we have provided a sequence of keepX of length: 4 for block OTUs and 14 for block Genes and 9 for block Metabolites.This results in 504 models being fitted for each component and each nrepeat.

```
list.keepX = tune$choice.keepX
DIABLOmod = block.splsda(X = data, Y = Y, ncomp = 1, design = design,keepX=list.keepX)

## Design matrix has changed to include Y; each block will be
##            linked to Y.

Step 4: DIABLO plots #A scatterplot displaying the first component in each data set (upper diago
nal plot) and Pearson correlation between components (lower diagonal plot).
MICEplotDiablo(DIABLOmod) ### Figure 5B

##
## Attaching package: 'ellipse'

## The following object is masked from 'package:graphics':
##
##     pairs
```

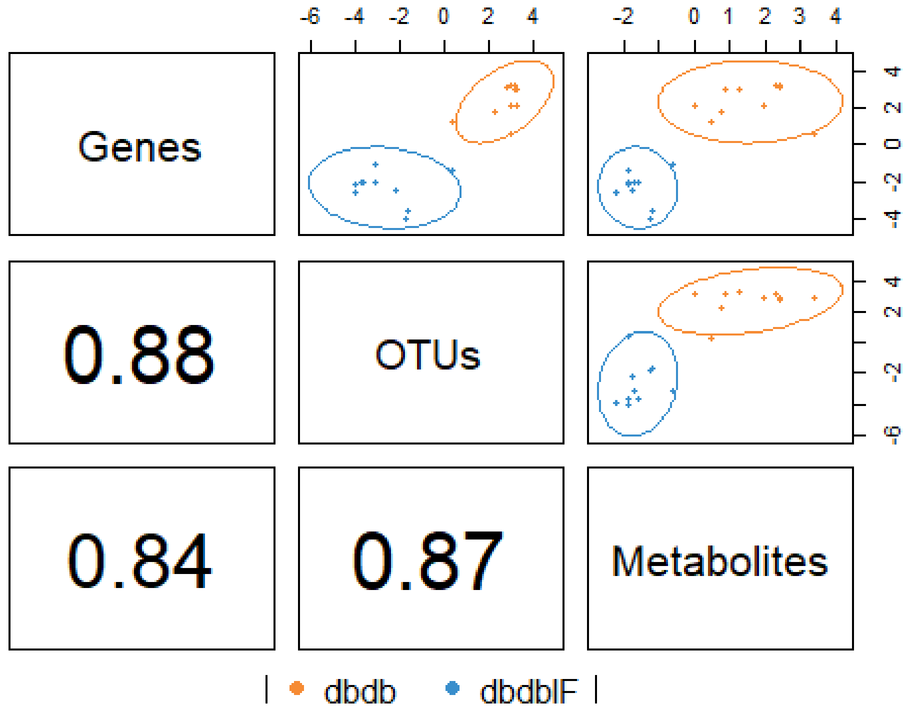


```
# The Circos plot shows the positive (negative) correlation, denoted as brown (grey) lines, between
 selected multi-omics predictors
circosPlot(DIABLOmod, cutoff = 0.4, line = FALSE,  color.blocks= c('gold','darkolivegreen2','red'),
         color.cor = c("chocolate3","grey20"), size.labels = 1.2,size.variables = 0.6)
```

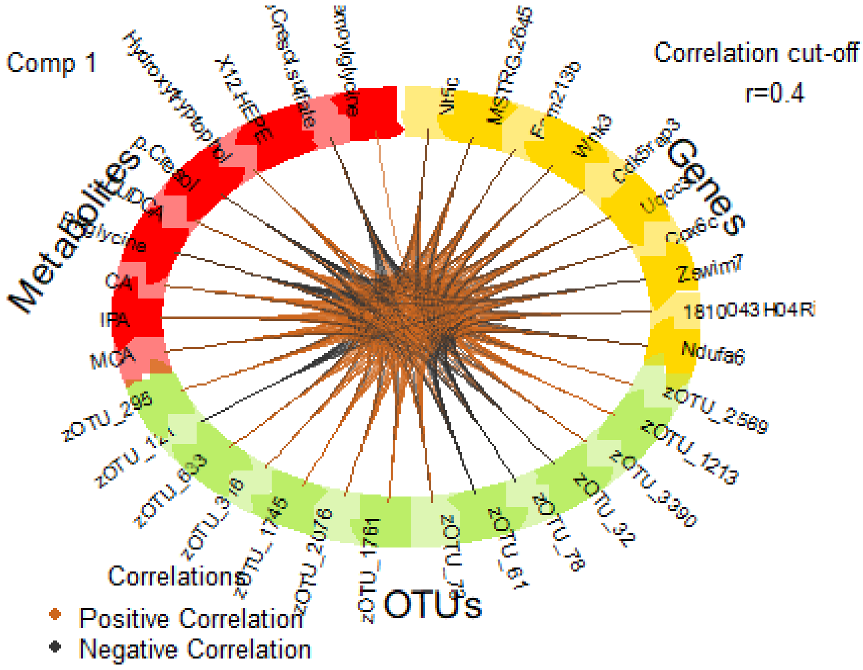


```
# A clustered Image Map (Euclidean distance, Complete linkage) of the multi-omics predictors. Samples are represented in rows, selected features on the first component in columns.

MICEcimDiablo(DIABLOmod, color.blocks = c('gold','darkolivegreen2','red'),comp = 1, margin=c(10,10), legend.position = "right",size.legend = 0.6,row.names =FALSE)
```

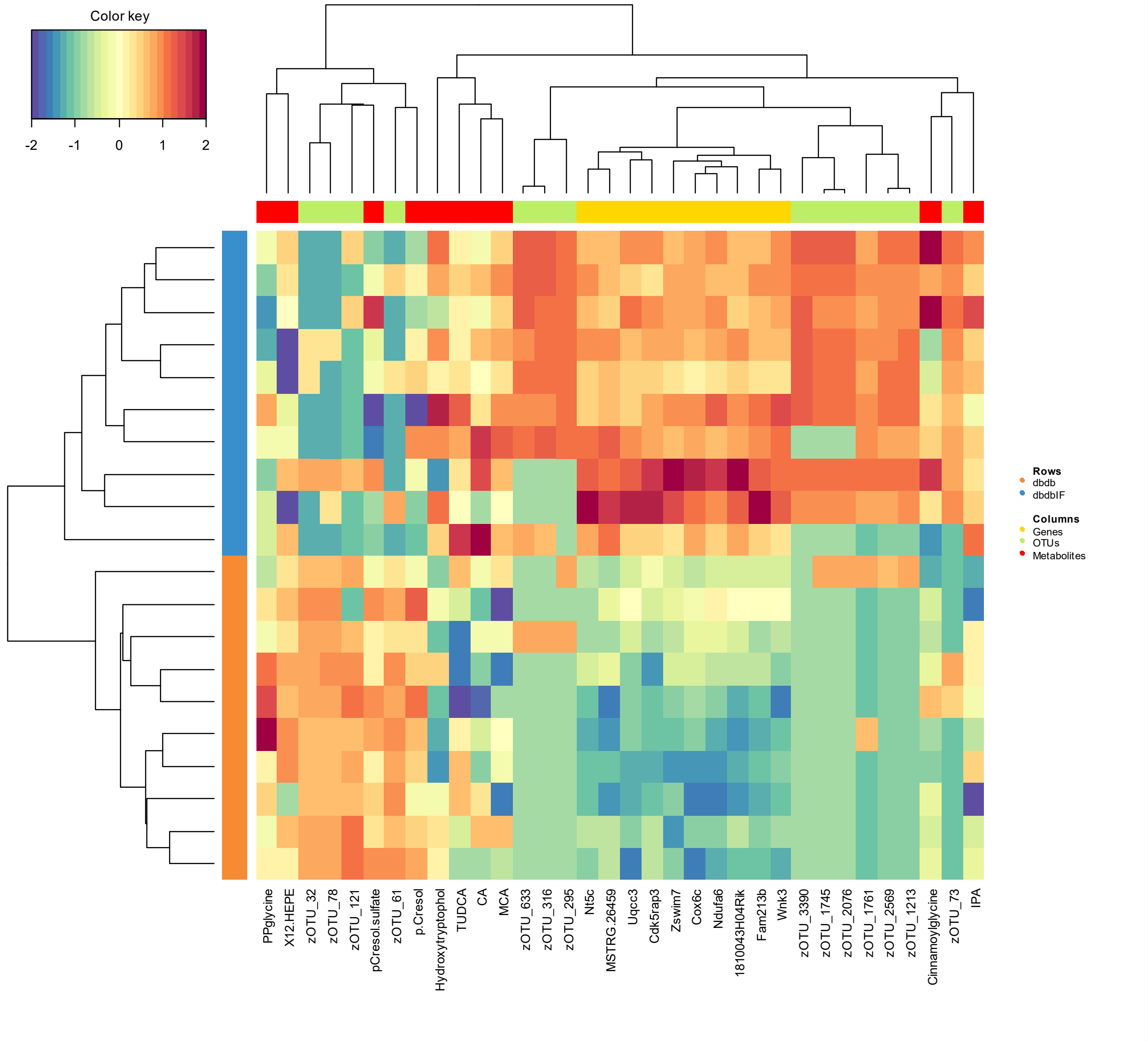


```
# loadings of multi-omics predictors selected for discriminating dbdb from dbdb-IF
par(mfrow = c(3, 1), # 2 x 2 pictures on one plot
    pty = "m",mar=c(4,3,4,2), par(cex.lab=2,cex=4) )
MICEplotLoadings(DIABLOmod, comp = 1, contrib = 'max', method = 'median',legend=FALSE,titl
e = "Genes", block = 'Genes')
MICEplotLoadings(DIABLOmod, comp = 1, contrib = 'max', method = 'median',legend=FALSE,titl
e = "OTUs", block = 'OTUs')
MICEplotLoadings(DIABLOmod, comp = 1, contrib = 'max', method = 'median',legend=FALSE,titl
e = "Metabolites", block = 'Metabolites')
```

Supplementary References

1. Bolger AM, Lohse M, Usadel B. Trimmomatic: a flexible trimmer for Illumina sequence data. *Bioinformatics* **30**, 2114-2120 (2014).

2. Kim D, Langmead B, Salzberg SL. HISAT: a fast spliced aligner with low memory requirements. *Nat. Methods* **12**, 357 (2015).

3. Pertea M, Pertea GM, Antonescu CM, Chang T-C, Mendell JT, Salzberg SL. StringTie enables improved reconstruction of a transcriptome from RNA-seq reads. *Nat. Biotechnol.* **33**, 290 (2015).

4. Frazee AC, Pertea G, Jaffe AE, Langmead B, Salzberg SL, Leek JT. Ballgown bridges the gap between transcriptome assembly and expression analysis. *Nat. Biotechnol.* **33**, 243 (2015).

5. Frazee AC, Pertea G, Jaffe AE, Langmead B, Salzberg SL, Leek JT. Flexible isoform-level differential expression analysis with Ballgown. *BioRxiv*, 003665 (2014).

6. Pertea M, Kim D, Pertea GM, Leek JT, Salzberg SL. Transcript-level expression analysis of RNA-seq experiments with HISAT, StringTie and Ballgown. *Nat. Protoc.* **11**, 1650 (2016).

7. Langfelder P, Horvath S. WGCNA: an R package for weighted correlation network analysis. *BMC bioinformatics* **9**, 559 (2008).

8. Shannon P*, et al.* Cytoscape: a software environment for integrated models of biomolecular interaction networks. *Genome Res.* **13**, 2498-2504 (2003).

9. Edgar R. UCHIME2: improved chimera prediction for amplicon sequencing. *BioRxiv*, 074252 (2016).

10. Rognes T, Flouri T, Nichols B, Quince C, Mahé F. VSEARCH: a versatile open source tool for metagenomics. *PeerJ* **4**, e2584 (2016).

11. Edgar R. UNOISE2: improved error-correction for Illumina 16S and ITS amplicon sequencing. BioRxiv. 2016: 081257. *Publisher Full Text*, (2016).

12. Nearing JT, Douglas GM, Comeau AM, Langille MG. Denoising the Denoisers: an independent evaluation of microbiome sequence error-correction approaches. *PeerJ* **6**, e5364 (2018).

13. DeSantis TZ*, et al.* Greengenes, a chimera-checked 16S rRNA gene database and workbench compatible with ARB. *Appl. Environ. Microb.* **72**, 5069-5072 (2006).

14. Caporaso JG*, et al.* QIIME allows analysis of high-throughput community sequencing data. *Nat. methods* **7**, 335 (2010).

15. Paulson JN, Stine OC, Bravo HC, Pop M. Differential abundance analysis for microbial marker-gene surveys. *Nat. Methods* **10**, 1200 (2013).

16. Dixon P. VEGAN, a package of R functions for community ecology. *J. Veg. Sci.* **14**, 927-930 (2003).

17. Lagkouvardos I, Fischer S, Kumar N, Clavel T. Rhea: a transparent and modular R pipeline for microbial profiling based on 16S rRNA gene amplicons. *PeerJ* **5**, e2836 (2017).

18. Mandal S, Van Treuren W, White RA, Eggesbø M, Knight R, Peddada SD. Analysis of composition of microbiomes: a novel method for studying microbial composition. *Microb. Ecol. Health. Dis* **26**, 27663 (2015).

19. Langille MG*, et al.* Predictive functional profiling of microbial communities using 16S rRNA marker gene sequences. *Nat. Biotechnol.* **31**, 814 (2013).

20. Edgar RC. Search and clustering orders of magnitude faster than BLAST. *Bioinformatics* **26**, 2460-2461 (2010).

21. Kanehisa M, Goto S. KEGG: kyoto encyclopedia of genes and genomes. *Nucleic acids Res.* **28**, 27-30 (2000).

22. Robinson MD, McCarthy DJ, Smyth GK. edgeR: a Bioconductor package for differential expression analysis of digital gene expression data. *Bioinformatics* **26**, 139-140 (2010).

23. Rowan S*, et al.* Involvement of a gut–retina axis in protection against dietary glycemia-induced age-related macular degeneration. *Proc. Natl. Acad. Sci. U.S.A.* **114**, E4472-E4481 (2017).

24. Shi L, Westerhuis JA, Rosén J, Landberg R, Brunius C. Variable selection and validation in multivariate modelling. *Bioinformatics* **35**, 972-980 (2018).

25. Filzmoser P, Liebmann B, Varmuza K. Repeated double cross validation. *J. Chemom. : A Journal of the Chemometrics Society* **23**, 160-171 (2009).

26. Varmuza K, Filzmoser P, Dehmer M. Multivariate linear QSPR/QSAR models: Rigorous evaluation of variable selection for PLS. *Comput. Struct. Biotechnol. J.* **5**, e201302007 (2013).

27. Pedersen HK*, et al.* A computational framework to integrate high-throughput '-omics' datasets for the identification of potential mechanistic links. *Nat. Protoc.* **13**, 2781 (2018).

28. Rohart F, Gautier B, Singh A, Lê Cao K-A. mixOmics: An R package for 'omics feature selection and multiple data integration. *PLoS Comput. Biol.* **13**, e1005752 (2017).

29. Shen X*, et al.* Normalization and integration of large-scale metabolomics data using support vector regression. *Metabolomics* **12**, 89 (2016).

30. Caporaso JG*, et al.* QIIME allows analysis of high-throughput community sequencing data. *Nat. Methods* **7**, 335-336 (2010).

31. Edgar RC. UNOISE2: improved error-correction for Illumina 16S and ITS amplicon sequencing. *BioRxiv*, 081257 (2016).
