# Supplementary material for: Gut microbiota mediates intermittent-fasting alleviation of diabetes-induced cognitive impairment
Source: Nat Commun. 2020 Feb 18;11:855. doi: 10.1038/s41467-020-14676-4 (PMC7029019; doi:10.1038/s41467-020-14676-4)
Supplement: Supplementary file 3 — Description of Additional Supplementary Files [file 41467_2020_14676_MOESM3_ESM.docx]

Description of Additional Supplementary Files

- File Name: **Supplementary Data 1**

Description: Summary of sequencing data from 31 samples

- File Name: **Supplementary Data 2**

Description: The FPKM value of 27,094 genes in 31 samples

- File Name: **Supplementary Data 3**

Description: All differentially expressed genes of 6 groups through DEG analysis

- File Name: **Supplementary Data 4**

Description: The GO terms of 49 hubgenes in WGCNA-brown module

- File Name: **Supplementary Data 5**

Description: The KEGG pathways of 49 hubgenes in WGCNA-brown module

- File Name: **Supplementary Data 6**

Description: Relative abundance of bacteria at different levels

- File Name: **Supplementary Data 7**

Description: Significant genera-phenotype correlation pairs (p < 0.05)

- File Name: **Supplementary Data 8**

Description: The KEGG pathway comparison between dbdbIF and dbdb on 28d with EdgR

- File Name: **Supplementary Data 9**

Description: 23 microbial derived metabolites whose levels were primarily modulated by the abundance of gut microbiota

- File Name: **Supplementary Data 10**

Description: Microbial derived metabolites concentration after antibiotic treatment
