# Supplementary material for: Gut microbiota mediates intermittent-fasting alleviation of diabetes-induced cognitive impairment
Source: Nat Commun. 2020 Feb 18;11:855. doi: 10.1038/s41467-020-14676-4 (PMC7029019; doi:10.1038/s41467-020-14676-4)
Supplement: Supplementary file 14 — Reporting Summary [file 41467_2020_14676_MOESM14_ESM.pdf]

nature research

Corresponding author(s): Zhigang Liu, Lin Shi, Shancen Zhao, and Xuebo Liu

Last updated by author(s): Jan 17, 2020

Reporting Summary

Nature Research wishes to improve the reproducibility of the work that we publish. This form provides structure for consistency and transparency in reporting. For further information on Nature Research policies, see Authors & Referees and the Editorial Policy Checklist .

Statistics

For all statistical analyses, confirm that the following items are present in the figure legend, table legend, main text, or Methods section.

n/a	Confirmed	
☐	☒	The exact sample size (*n*) for each experimental group/condition, given as a discrete number and unit of measurement
☐	☒	A statement on whether measurements were taken from distinct samples or whether the same sample was measured repeatedly
☐	☒	The statistical test(s) used AND whether they are one- or two-sided<br>*Only common tests should be described solely by name; describe more complex techniques in the Methods section.*
☐	☒	A description of all covariates tested
☐	☒	A description of any assumptions or corrections, such as tests of normality and adjustment for multiple comparisons
☐	☒	A full description of the statistical parameters including central tendency (e.g. means) or other basic estimates (e.g. regression coefficient) AND variation (e.g. standard deviation) or associated estimates of uncertainty (e.g. confidence intervals)
☐	☒	For null hypothesis testing, the test statistic (e.g. *F*, *t*, *r*) with confidence intervals, effect sizes, degrees of freedom and *P* value noted<br>*Give P values as exact values whenever suitable.*
☒	☐	For Bayesian analysis, information on the choice of priors and Markov chain Monte Carlo settings
☐	☒	For hierarchical and complex designs, identification of the appropriate level for tests and full reporting of outcomes
☐	☒	Estimates of effect sizes (e.g. Cohen's *d*, Pearson's *r*), indicating how they were calculated

Our web collection on statistics for biologists contains articles on many of the points above.

Software and code

Policy information about availability of computer code

Data collection	All the software used in current study for data collection are commercial. Detailed information are provided in the text and in supplemental methods. No custom code has been used .
Data analysis	All the software and codes used in current study are ether commercial or have been previously published. Detailed information are provided in the text and in supplemental methods. No custom code has been developed and applied in current study .

For manuscripts utilizing custom algorithms or software that are central to the research but not yet described in published literature, software must be made available to editors/reviewers. We strongly encourage code deposition in a community repository (e.g. GitHub). See the Nature Research guidelines for submitting code & software for further information.

Data

Policy information about availability of data

All manuscripts must include a data availability statement. This statement should provide the following information, where applicable:

- Accession codes, unique identifiers, or web links for publicly available datasets
- A list of figures that have associated raw data
- A description of any restrictions on data availability

The accession number for the entire RNA-seq dataset reported in this manuscript is GSE125387. Here is the link for reviewers to access this dataset: ftp://ftp-trace.ncbi.nlm.nih.gov/sra/review/SRP181024_20190131_155134_305cf1fb13b9539dcd317a0354c9ed61
The accession number for the entire 16S rRNA sequencing dataset reported in this manuscript is SRP181000. Here is the link for reviewers to access this dataset: ftp://ftp-trace.ncbi.nlm.nih.gov/sra/review/SRP181000_20190129_081421_6a55596c15df4993eea3b44eace1ee7f
The RNA-seq and 16S rRNA sequencing data that support the findings of this study have been also deposited in the CNSA (https://db.cngb.org/cnsa/) of CNGBdb with accession number CNP0000608.
Supplemental tables contain an Excel file of RNA-sequencing, 16S rRNA sequencing, and metabolome data. Other raw data (for editor and reviewers) were deposited in figshare dataset as follows:

https://figshare.com/s/58ccac4aa614dd4ade84

Field-specific reporting

Please select the one below that is the best fit for your research. If you are not sure, read the appropriate sections before making your selection.

- [x] Life sciences
- [ ] Behavioural & social sciences
- [ ] Ecological, evolutionary & environmental sciences

For a reference copy of the document with all sections, see nature.com/documents/nr-reporting-summary-flat.pdf

Life sciences study design

All studies must disclose on these points even when the disclosure is negative.

Sample size	There are four sets of animal study in current research. The sample size were 7-11 in these experiments. For the Sequencing/OMICs studies in current research, the sample size was 9-11 based on the quality of the tissue/blood/fecal samples. These sample sizes was chosen based on the previous research and the sizes are sufficient.
Data exclusions	The details of data exclusion has been provided in the Methods.
Replication	It is confirmed that the benefits of IF regimen on biological and behavioral disorder in db/db mice could be successfully replicated when follow the description of the method section.
Randomization	Diabetic animals were randomly divided into several group for IF, antibiotic or metabolites treatment.
Blinding	No blinding for the animal experiments as the phenotype of the diabetic animal are apparently distinguished from non-diabetic ones.

Reporting for specific materials, systems and methods

We require information from authors about some types of materials, experimental systems and methods used in many studies. Here, indicate whether each material, system or method listed is relevant to your study. If you are not sure if a list item applies to your research, read the appropriate section before selecting a response.

Materials & experimental systems

n/a	Involved in the study	
[ ]	[x]	Antibodies
[x]	[ ]	Eukaryotic cell lines
[x]	[ ]	Palaeontology
[ ]	[x]	Animals and other organisms
[x]	[ ]	Human research participants
[x]	[ ]	Clinical data

Methods

n/a	Involved in the study	
[x]	[ ]	ChIP-seq
[x]	[ ]	Flow cytometry
[x]	[ ]	MRI-based neuroimaging

Antibodies

Antibodies used	We described all the antibodies used in the study with supplier name and identifiers in the supplemental table.
Validation	The source/manufacurer's name of all primary antibodies were provided in the supplemental table.

Animals and other organisms

Policy information about studies involving animals; ARRIVE guidelines recommended for reporting animal research

Laboratory animals	Male BKS.Cg-Dock7m+/+ Leprdb/J (Stock No: 000642) Homozygous Leprdb/db mice were diabetic, and heterozygous Leprdb/m mice were used as controls (denoted as db/db and db/m in the text) in the present study. Age: 4 months.
Wild animals	The study did not involve samples collected from the field.
Field-collected samples	The study did not involve samples collected from the field.
Ethics oversight	All of the experimental procedures were followed using the Guide for the Care and Use of Laboratory Animals: Eighth Edition (ISBN-10: 0-309-15396-4) and protocols were approved by the Northwest A&F University, and BGI Institutional Review Board on Bioethics and Biosafety (BGI-IRB).

Note that full information on the approval of the study protocol must also be provided in the manuscript.
